# Supplementary material for: annoFuse: an R Package to annotate, prioritize, and interactively explore putative oncogenic RNA fusions
Source: BMC Bioinformatics. 2020 Dec 14;21:577. doi: 10.1186/s12859-020-03922-7 (PMC7737294; doi:10.1186/s12859-020-03922-7)
Supplement: Supplementary file 1 — Additional file 1: Figure S1. Fusions found in more than 1 histology. Barplots represent the number of histologies in which each fusion was observed. Dotted line represents the cut off (> 4 counts) used to remove potential false positives and fusions containing pseudogenes. Figure S2. Distribution of spanningDelta for annoFuse prioritized fusions from TCGA and PBTA cohorts. The spanningFragCountFilter and mean are plotted for each filtering cutoff of 10, 20, 30, 40, 50, 100, 150, and 200 spanningDelta for TCGA (N = 160 samples) (A) and PBTA (N = 1028 samples) (B) fusions. Figure S3. Sensitivity of TCGA fusions retained by annoFuse. At a spanningDelta of 100, annoFuse achieved a sensitivity of 96.35% for fusions in the TCGA final call set. The x-axis represents the cutoff for spanningFragCountFilter used and y-axis represents the sensitivity of the fusions retained after fusion_filtering_QC. Figure S4. Distribution of kinase genes fused in 5' and 3' genes per histologies. For each broad histology, pie charts represent the percentage of fusions in which Gene1A (5’) or Gene1B (3’) retain their kinase domains. [file 12859_2020_3922_MOESM1_ESM.pdf]

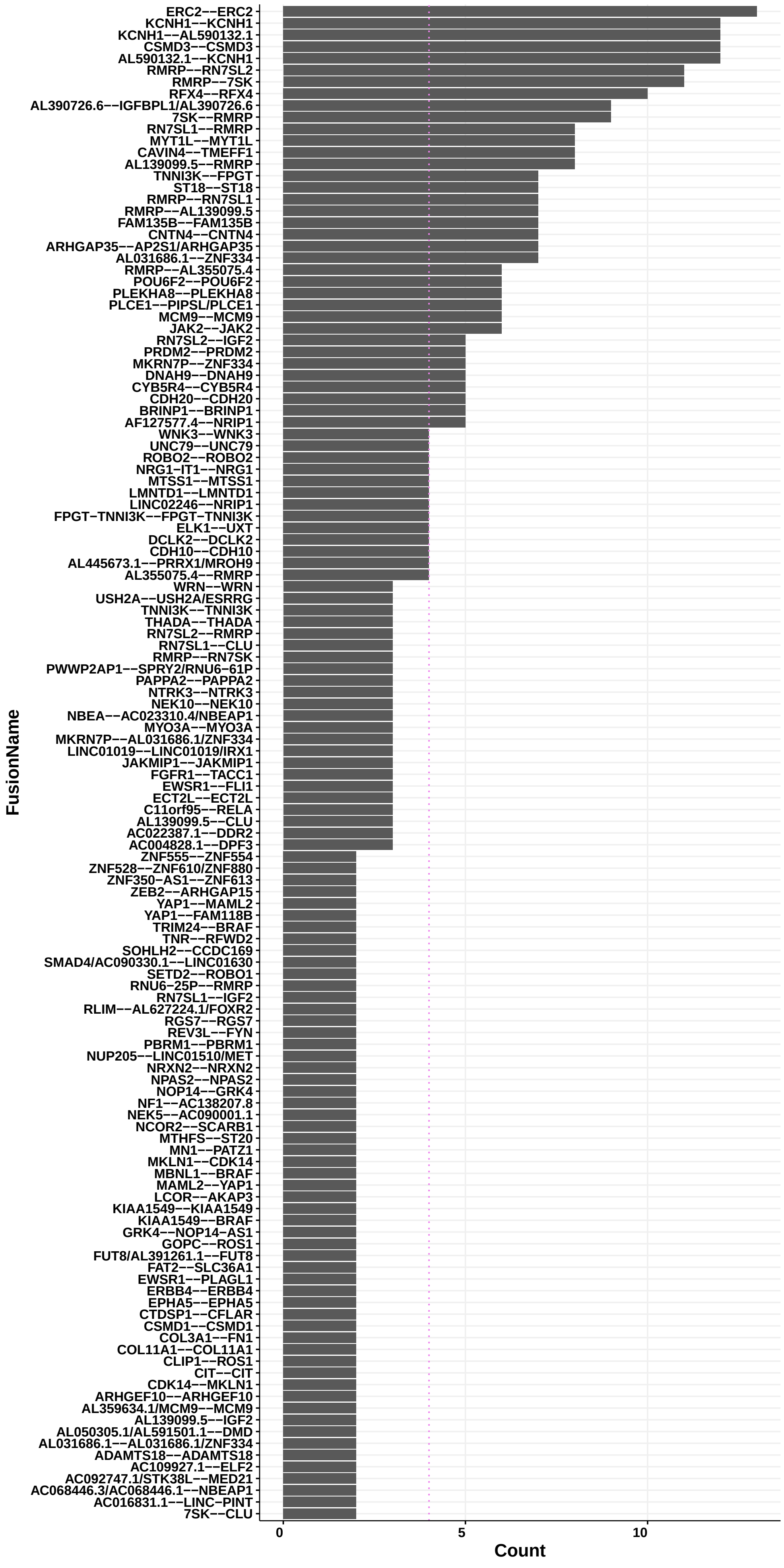

Figure S1: Fusions found in more than 1 histology

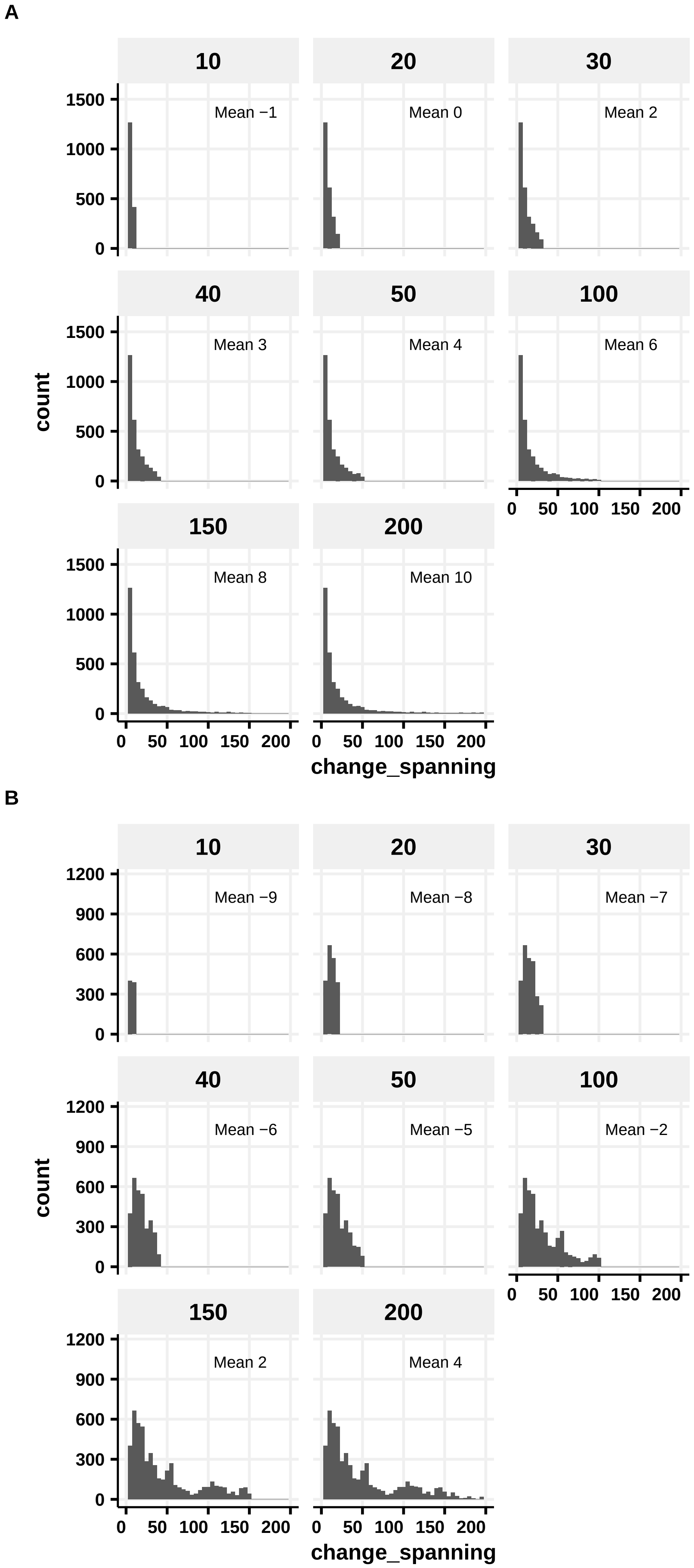

Figure S2: Distribution of spanningDelta for annoFuse prioritized fusions from TCGA and PBTA cohorts

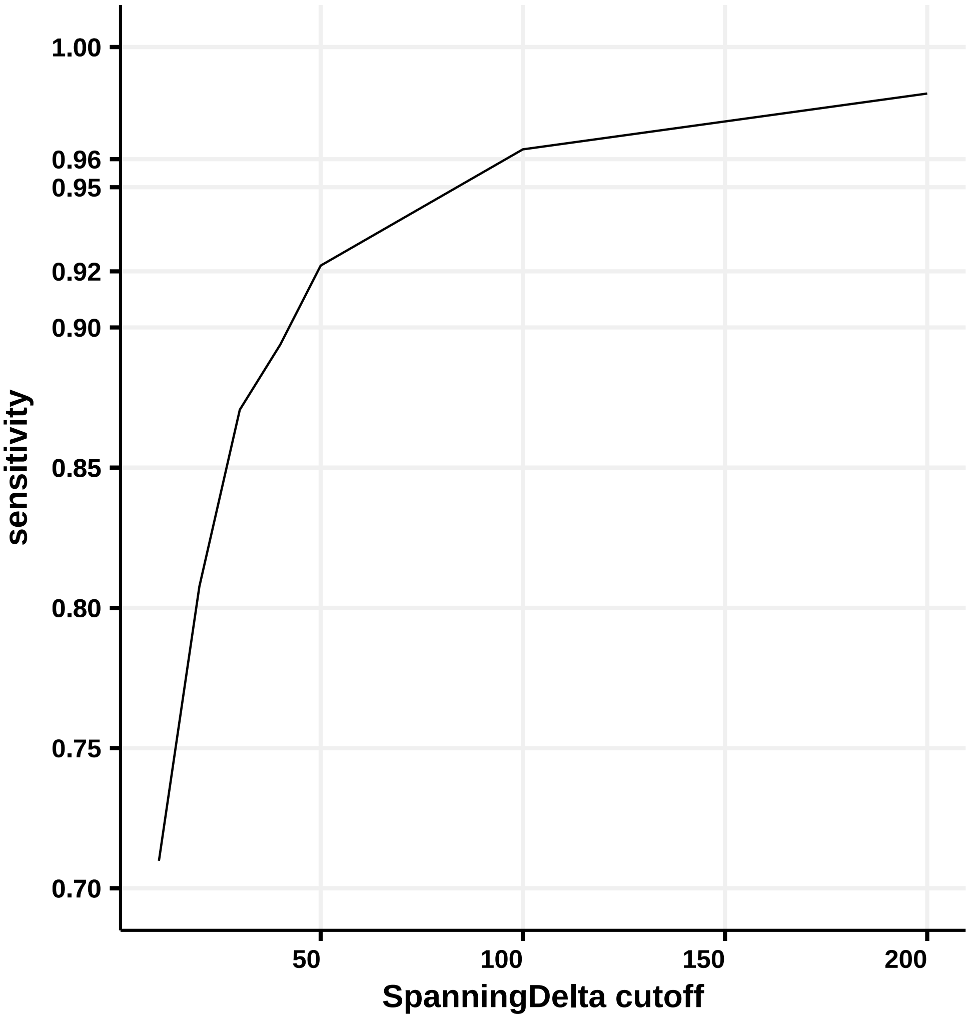

Figure S3: Sensitivity of TCGA fusions retained by annoFuse

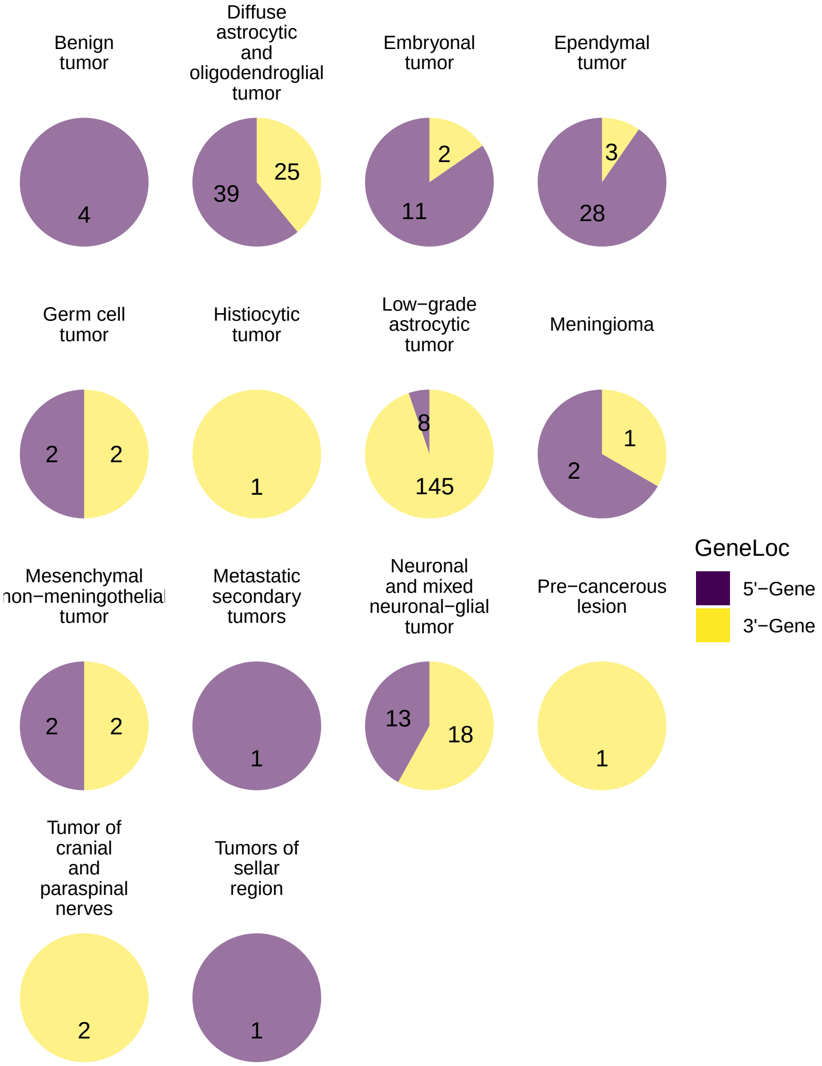

Figure S4: Distribution of kinase genes fused in 5' and 3' genes per histologies
